# Development and Experimental Verification of Inorganic Electromagnetic Pulse Shielding Paint for Building Interiors Using Carbon-Based Materials

**DOI:** 10.3390/ma17122863

**Published:** 2024-06-12

**Authors:** Kyong-Pil Jang

**Affiliations:** Department of Building Research, Korea Institute of Civil Engineering and Building Technology, (Daehwa-Dong) 283, Goyangdae-ro, Ilsanseo-gu, Goyang-si 10223, Gyeonggi-do, Republic of Korea; kyongpiljang@kict.re.kr

**Keywords:** electromagnetic pulse, shielding effectiveness, inorganic, paint, carbon material

## Abstract

The term electromagnetic pulse (EMP) generally refers to high-power electromagnetic waves and can be classified into EMPs caused by nuclear weapons, non-nuclear EMPs, and EMPs caused by natural phenomena. EMPs can cause catastrophic damage to any electronic device consisting of electromagnetic components, including communications devices and transportation. In this study, the shielding effectiveness of paint was evaluated depending on the type and content of carbon material and binder. To analyze the compatibility and dispersibility improvement of the raw materials used in paint manufacturing, experiments were conducted in two stages, using 27 mixtures. The shielding effectiveness was evaluated for the optimal mixture developed through mixture experiments. The results of this study confirmed that the developed EMP shielding paint can improve the shielding effectiveness of concrete by 25–40 dB. Additionally, the adhesion strength and moisture resistance evaluation of the EMP shielding paint were evaluated. The average adhesive strength of the EMP shielding paint was 1.26 MPa. In moisture-resistance testing at a temperature of 50 ± 3 °C and a relative humidity of 95% or higher for more than 120 h, no cracks or peeling were observed on the painted surface.

## 1. Introduction

### 1.1. Background and Purpose

The damage caused by electromagnetic pulses (EMPs) was first discovered during nuclear weapon experiments conducted by the United States in the early 1960s in Pacific regions [1]. Since then, extensive research has been conducted on EMP shielding. EMPs have been found to cause malfunctions and faults in electronic communication systems, radios, televisions, and other electronic devices [2,3]. Moreover, repairing these problems can be time-consuming and costly. It has been found that most buildings in South Korea do not have EMP protection capabilities, and there is also a lack of standards for the installation of EMP protection facilities [4]. As today’s society continues to evolve into an era of information and digitalization, not only are important social infrastructure facilities interconnected, but so are the personal communication devices used by individuals. To prevent damage caused by high-power EMPs in these situations, additional research on EMP shielding is necessary.

EMP shielding refers to a phenomenon in which electromagnetic pulses traveling in the air encounter a shielding material and go through absorption and reflection processes, causing loss or extinction of electromagnetic pulse intensity [5,6,7,8]. Absorption means converting radio pulse energy into another form, such as heat energy or electric current. When radiated electromagnetic pulses reach an electromagnetic absorber, some are reflected and most are transmitted, and radio pulses of a certain frequency are converted into heat energy in the absorber. The intensity of radio pulses is weakened in proportion to the amount of converted energy, and materials that play this role are called radio pulse absorbers. Radio pulse absorbers include pyramid absorbers, ferrite tiles, and ferrite rubber sheets. The reflection is a shielding method that does not absorb radio pulses but reflects them at the same angle as the angle of incidence. The shielding method using reflection is generally used to shield high frequencies, and can be implemented using materials with high conductivity such as copper or aluminum.

To provide EMP shielding for buildings, the inherent shielding effectiveness of materials and design and construction techniques for the shielding structure are required. In particular, in the case of information and communication infrastructure that has a significant impact on national security and the economy and society, there are many facilities that have already been built and are in operation rather than new ones. When new buildings are constructed, it is important to include EMP protection at the design stage by using materials (such as EMP-shielding concrete and conductive metal plates) and components (such as shielding doors and windows) that have shielding capabilities. However, for existing facilities, it is more efficient from the perspective of spatial utilization and cost to apply coating treatments based on paint [9]. 

The specifications for the shielding effectiveness and physical properties of EMP shielding inorganic paint for building interiors have recently been established [10]. The EMP shielding inorganic paint that is developed in this study can be considered a paint with EMP shielding capabilities added to a commonly used water-based paint. To develop an EMP shielding inorganic paint in this study, mixture experiments were performed using various shielding materials and binders. The shielding materials and binders optimized for the EMP shielding inorganic paint were selected based on an EMP shielding effectiveness evaluation.

### 1.2. Objectives

EMP shielding methods can be classified into two categories: those that utilize metallic materials and those that utilize carbon-based materials [11,12,13,14,15]. The objective of this study is to develop an EMP shielding inorganic paint. Metal materials are not suitable for paint, owing to their high specific gravity and susceptibility to corrosion. Therefore, carbon-based materials were selected as shielding raw materials, and the EMP shielding effectiveness according to the type and amount of carbon materials used was evaluated to derive the optimal mix for EMP shielding paint. The shielding effectiveness represents the degree of EMP shielding expressed in decibels (dB). The target EMP shielding effectiveness set in the initial experimental stage was 20 dB within the frequency range of 1.5 GHz, whereas the ultimate development goal was to exceed 40 dB as specified in the KTL L-378-2022 [10] standard.

### 1.3. Research Trends

Carbon materials, such as carbon nanotubes (CNTs), graphite, carbon black, acetylene black, and carbon fiber, are commonly used in EMP-shielding inorganic paints [16,17,18]. In addition, to improve shielding performance, paints are often created by mixing paint with metal materials that conduct electricity well, such as copper and nickel [19]. Recently, a new type of nanomaterial transition metal called MXene has been developed [20,21]. This nanomaterial has a high EMP shielding effectiveness that exceeds 60 dB at a thickness of approximately 10 μm [20]. In addition to shielding paint, a technique to shield against EMPs by coating concrete surfaces with metal spraying has recently been developed [22].

Aside from shielding with paint and coatings, there are ongoing developments in the field of concrete mixtures that can provide EMP-shielding capabilities to structures, such as EMP shielding concrete and EMP shielding cement composites [23]. Lee et al. (2019) [4] evaluated the EMP shielding effectiveness of high-performance cement composites by examining electrical conductivity, EMP shielding experiments, and mechanical performance. They reported that the addition of carbon black and steel slag effectively enhanced the shielding effectiveness of cement composites. Min et al. (2019) [24] and Min (2020) [25] conducted research using metal-based industrial byproducts as aggregates for EMP shielding concrete. They found that electric arc furnace oxidizing and copper slag have positive effects on the shielding effectiveness. Min and Cho (2022) [26] evaluated the shielding effectiveness of EMP shielding concrete at various levels of milled carbon content. It was reported that the greatest improvement in shielding effectiveness occurred when the milled carbon content was 5%, and the shielding effectiveness increased at increasing concrete thickness [26].

A review of previous research on EMP shielding technology and materials indicated that there is still insufficient research on the development of EMP shielding paints for buildings [16,17,18,19,20,21,22,23,24,25,26]. Using EMP shielding concrete alone has limitations in shielding the entire structure, and the use of metal coating technology for the interior of buildings requires special equipment and high temperatures, making it more challenging than painting in terms of pretreatment and construction methods [22,23,24,25,26]. This study aimed to develop an EMP shielding inorganic paint applicable to buildings and to derive the optimal mixture based on various mixture experiments.

## 2. Experiments and Methods

### 2.1. Experimental Overview

The paint consists of a film-forming element (binder), which is the main component of the paint film; a film-forming minor element (additive) used to improve the performance of the paint film; pigments for color; and a paint-forming auxiliary element (solvent) used to dissolve the binder.

This study conducted experiments to select the binder and pigment components of paint to develop EMP shielding inorganic paint. The pigment in the shielding paint is a shielding material that blocks EMPs. As described in Section 1.3, the shielding materials used in paint include CNTs, graphite, carbon black, acetylene black, and carbon fiber. Among these, CNTs are more expensive compared with other materials owing to their unique refining and synthesis methods and are difficult to mass-produce for paint manufacturing.

In the case of carbon fiber, since it is in the form of needle-shaped, homogeneity cannot be guaranteed even after a dispersion process, and the construction performance of the paint may be reduced. Therefore, this study selected three carbon materials, namely graphite (GK, SC 5), acetylene black (Hexing, HX-501), and carbon black (Unipetrol, AC-60), as shielding materials because they are easily available and have uniform particle sizes.

Four types of binders were used: sodium silicate (YI, No. 3), potassium silicate (YI, PS-C300), inorganic binder (YI, SFR-0582), and nanohybrid resins (YI, NH-921-3). The binder was selected among three types of inorganic materials that have few to no volatile organic compounds or harmful substances based on considerations of their environmental friendliness. Furthermore, an additional type that was a mixture of organic and inorganic materials was used for performance comparisons.

The experiment was conducted with two experimental groups. The experiment for the first group involved the selection of the most suitable binder for the shielding paint. The paint mixture experiments were conducted by changing the binder type while keeping the shielding material type consistent. The second experiment involved the identification of the optimal mixture proportion of shielding materials. The second mixture experiments were conducted using only the binder selected in the first experiment while varying the amounts of the shielding materials.

### 2.2. Used Materials

Three types of carbon material were used as shielding materials in this study: graphite, acetylene black, and carbon black. The main physical properties of the shielding materials used in this study are presented in Table 1. For all three materials, high-purity products with carbon contents of >95% were used. The average particle size ranged from 2.0 μm to 7.0 μm for graphite, was 35 nm for acetylene black, and ranged from 6.5 μm to 12.5 μm for carbon black.

The four types of materials used as binders in this study are (as mentioned before) sodium-based water glass, potassium-based water glass, inorganic binder, and nano-hybrid resin. The specifications of the binders used in this study are presented in Table 2. Soluble sodium silicate, the formal name for water glass, is an inorganic compound composed of silica (SiO_2_) and alkali metal (M_2_O) at various molar ratios. It is also known as water glass owing to its solubility in water. Inorganic binder is a modified form of potassium-based and sodium-based water glass, which is a fire-resistant inorganic material based on modified silicates. It is commonly used in the formation of mineral boards, sandwich panels, and the shaping of components that require high-temperature resistance. Nanohybrid resin is an aqueous organic–inorganic hybrid resin with a high silica content with improved heat resistance and physicochemical properties compared with those of organic resins. It is mainly used in the manufacturing of functional coatings and paints that require heat resistance and physicochemical properties.

### 2.3. Paint Manufacturing and Experimental Details

**Figure 1:**
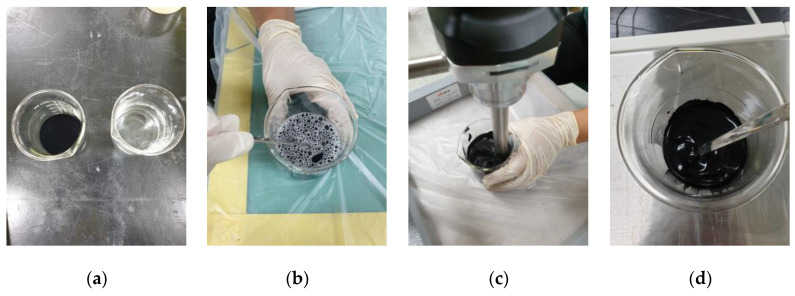
Manufacturing process of inorganic shielding electromagnetic pulse (EMP) paint: (**a**) material preparation and weighing, (**b**) hand mixing, (**c**) homogenizing, and (**d**) complete product.

In this study, graphite, acetylene black, and carbon black were utilized as the primary shielding materials for the development of EMP shielding paint. Sodium-based water glass, potassium-based water glass, inorganic binder, and nanohybrid resin were used as binders. Figure 1 shows the paint mixing process. After measuring the shielding material, water, and binder, they were mixed for approximately 5 min using an experimental reagent spoon. The mixture was then homogenized using a homogenizer to complete the mixing process. Carbon black is hydrophobic and does not readily mix with water. Therefore, a small amount of surfactant was added and then stirred using a homogenizer for approximately 5 to 10 min to achieve a well-dispersed condition.

**Figure 2:**
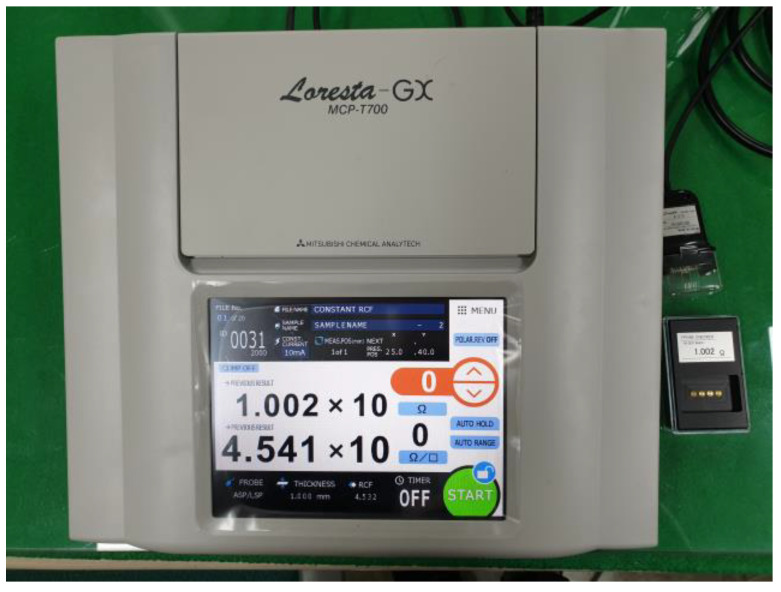
Electrical resistance measuring equipment.

The binders and shielding materials were selected based on their electrical resistance values. For each mixture, the electrical resistance values were measured, and materials with lower resistance values were chosen as the based raw materials for EMP shielding paint. The resistance measurement was conducted using the Loresta-GX MCP-T700 and the process of measuring electrical resistance using the equipment is illustrated in Figure 2. The EMP shielding effectiveness was measured using the experimental method specified in the American Society for Testing and Materials (ASTM) standard D 4935 [27]. Owing to the difficulties associated with consistent production and measurements of specimens that meet the specifications for many mixtures, a relative comparison was conducted based on electrical resistance. EMP shielding effectiveness is proportional to electrical conductivity [28]. In other words, high EMP shielding effectiveness means that the electrical resistance is low and the current flows well. Therefore, a simple measurement of electrical resistance was performed to determine the optimal mixture, and an evaluation of the shielding effectiveness was then conducted.

The resistance measurements with the Loresta-BX MCP-T700 equipment used a 4-terminal method (JIS K 7194 [29]) to compensate for the resistance of the measurement cables and internal resistance, unlike a conventional resistance tester. Errors in the measured value occur depending on how the resistivity correction factor (RCF) is handled. The resistance measurement equipment used in this study achieved high reliability by optimizing the RCF value based on Poisson’s equation and performing calibrations according to the measurement position.

**Figure 3:**
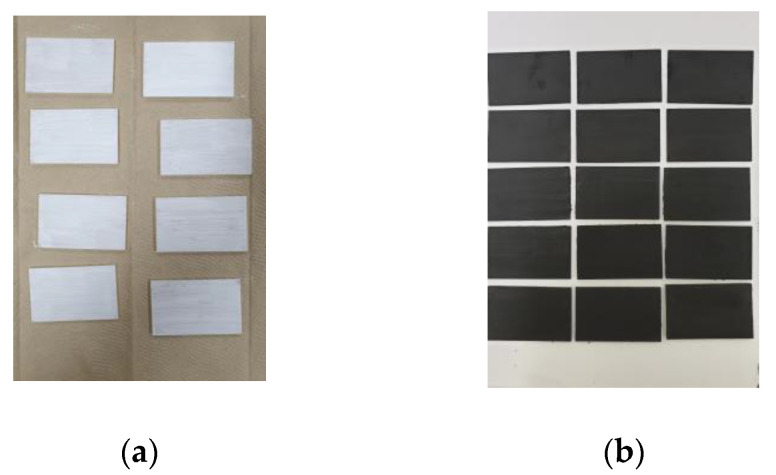
Specimen manufacturing process for electrical resistance evaluation: (**a**) Gesso-coated specimen and (**b**) inorganic EMP shielding paint-coated specimen.

To measure electrical resistance, according to the JIS K 7194 standard [29], acrylic specimens in the shape of a rectangle measuring 5 cm × 8 cm were fabricated for each mixture, as shown in Figure 3. The acrylic surface of the specimen was pretreated with gesso, and then four coats of the mixed paint were applied over it. The reason for pretreating with gesso is that the direct application of water-based paint to an acrylic surface degrades its adhesion.

**Figure 4:**
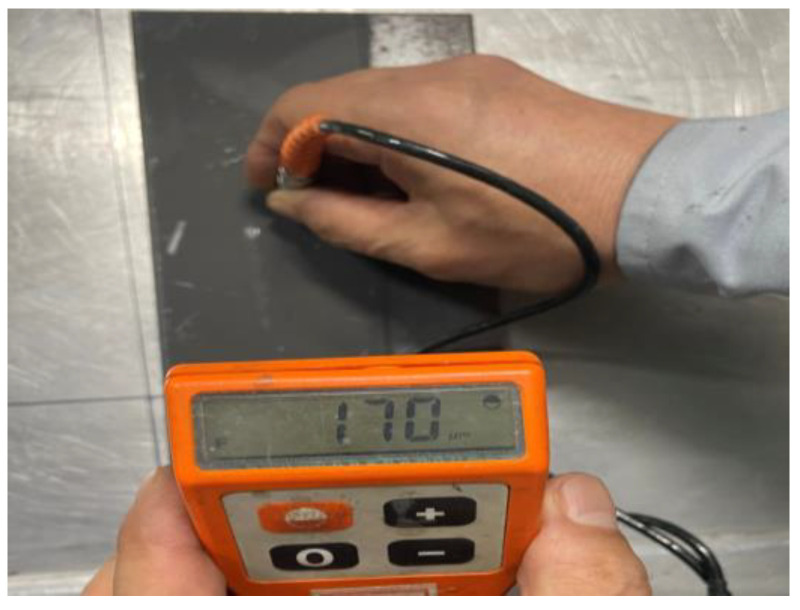
Measurement of the coated paint thickness.

Four specimens were fabricated at each composition setting, and the thickness and electrical resistance were measured after each coating process. The thickness of each coating layer was approximately 50 μm when applied once, and the total thickness after four coatings was approximately 200 μm. Figure 4 illustrates the process of measuring the coating thickness. The thickness of the acrylic specimen could not be measured using the equipment because it was designed specifically for the measurement of metal substrates. Therefore, the thickness was measured by applying a coating to a metal substrate under the same conditions.

Painting the interior of a building requires the application of paint to concrete or plasterboard surfaces. Thus, it is preferable to use cellulose-reinforced cement (CRC) or gypsum board specimens when conducting experiments. However, the inhomogeneity of the surface of the CRC board can influence the results of electrical resistance measurements, while the needle-shaped probe of the measurement equipment may damage the gypsum board. Therefore, acrylic specimens with high surface homogeneity and strength were used. Furthermore, it was determined that conducting experiments using acrylic specimens was not only more efficient in terms of specimen preparation but also more cost-effective compared with purchasing boards for cutting or ordering custom-made specimens for small-scale experiments. Hence, it was decided to conduct the experiments using acrylic specimens.

## 3. Mixing Experiments and Results of Inorganic EMP Shielding Paint

### 3.1. First Mixing Experiment and Results

In this study, two sets of mixing experiments were conducted. Experiment 1 was conducted to select the most suitable binder for the shielding paint. This experiment involved changing the binder type while keeping the same type of shielding material. Experiment 2 aimed to find the optimal mixture proportion of shielding materials. Only the binder selected in the first experiment was used while varying the amounts of the shielding materials.

Table 3 shows the mixture (mix) results for selecting the most suitable binder for the shielding paint. The shielding material utilized was graphite, which readily dissolves in water and water glass. This experiment was conducted by maintaining the same amount of graphite and altering the type and amount of binder (binder content relative to graphite weight 0.5, 1.0, 1.5, and 2.0).

The experiments conducted from mix 1-1 to mix 1-5 (see Table 3) involved the mixing of water and four types of binders with graphite at a 1:1 ratio. These experiments were performed as a preliminary experiment to assess the fluidity, spreadability, and other properties of the paint. The electrical resistance values listed in Table 3 are measurements acquired at a thickness of 200 μm after the deposition of four coatings. The measurements were conducted at the centers of the specimens where the coating surface was most uniform and exhibited minor clustering effects. This was uniformly applied to all mixture experiments.

A review of the results of the electrical resistance measurements of the mixtures conducted as part of the first experiment revealed that the mixture using nanohybrid resin showed the highest electrical resistance value of 64.5 Ω. The mixture using sodium silicate had the second highest resistance at 7.91 Ω, followed by water-only at 5.54 Ω, and potassium silicate at 3.43 Ω. The lowest resistance was measured at 2.22 Ω when the inorganic binder was used.

The electrical resistance value could be measured in a basic experiment mixing graphite and binder in a 1:1 ratio. However, the paint’s state was almost non-fluid, and it was not easily spreadable with a brush. Therefore, in the experiment of measuring electrical resistance according to the type and content of the binder, water was added in addition to the binder (an amount of 0.7 times the weight of graphite was used) to ensure fluidity and spreadability. In the second experiment, the nanohybrid resin binder with the highest resistance value measured in the first experiment was excluded. Nanohybrid resin is a binder that combines silica and organic compounds. Compared with other binders used in this study, it had a higher solid content, which ranged from approximately 5% to a maximum of 8%. Therefore, even when the same amount was used, the proportion of shielding material in the paint was lower compared with those of other mixtures, resulting in a considerably higher resistance value.

**Figure 5:**
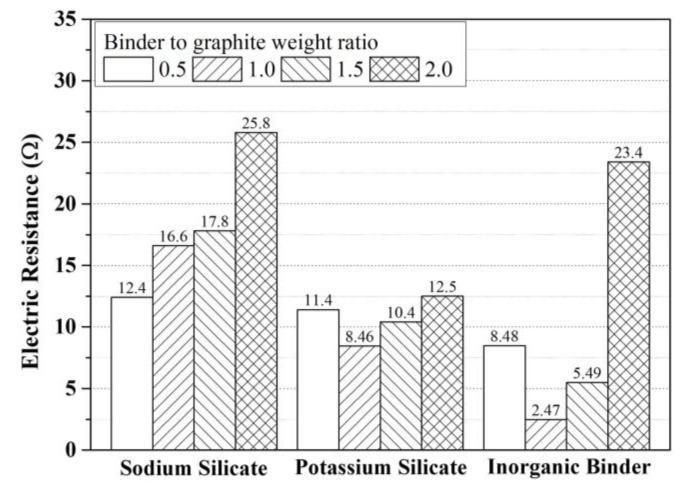
Electrical resistance results according to binder type and dosage.

The electrical resistance measurements of paints mixed with binder contents at the ratios of 0.5, 1.0, 1.5, and 2.0 relative to the weight of graphite are presented in Figure 5. In general, the measured electrical resistance values of the paint using sodium silicate were found to be high.

In mixtures using potassium silicate, the ratio of graphite to the binder at 1:1 resulted in the lowest electrical resistance value of 8.46 Ω. Subsequently, an increase in the binder’s content led to an increasing electrical resistance tendency.

In inorganic binder mixtures, the lowest measured electrical resistance of 2.47 Ω was observed when the ratio of graphite to binder was 1:1. Subsequently, an increase in the amount of binder resulted in an increasing trend in electrical resistance values.

The mixture experiment for binder selection confirmed that modifying sodium silicate and potassium silicate to create an inorganic binder had a positive effect that improved the shielding effectiveness of the paint compared with the outcomes obtained when these were used alone.

### 3.2. Second Mixing Experiment and Results

The most effective binder for improving the EMP shielding effectiveness of paint was selected based on the first mixing experiment. The purpose of the second experiment was to determine the optimal mixture of shielding materials using the binder selected in the first mixing experiment. The mixing experiment was conducted by mixing graphite as the main shielding material with acetylene and carbon black as additives, as listed in Table 4. The mixing ratios of acetylene and carbon black relative to the weight of graphite were set at 0.1, 0.15, 0.2, 0.25, and 0.3. The particle sizes of acetylene black and carbon black were on average 0.035 μm and 9 μm, respectively. They had densities of 250 g/L and 112 g/L, making them extremely lightweight, and they possessed insolubility in water. Therefore, it was necessary to mix water and a surfactant in a solution and perform a process of dispersion and homogenization. The mixture was obtained by increasing the water amount based on the content of acetylene and carbon black.

**Figure 6:**
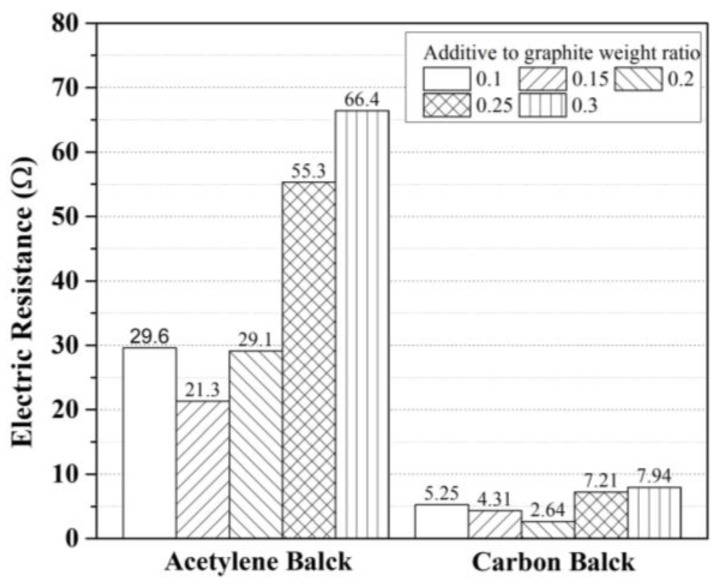
Results of electrical resistance according to additive material type and dosage.

Figure 6 shows the measured electrical resistance values of inorganic EMP shielding paint using acetylene and carbon black. The resistance measurement results for different types of materials showed that carbon black exhibited significantly lower resistance values compared with those of acetylene black. Acetylene black is a widely used additive used in electronics, such as batteries and power cables, owing to its high electrical conductivity. However, owing to its insolubility in water, it requires the use of appropriate surfactants and dispersants that match the material’s properties when used as an additive in paint. The reason for the higher resistance values observed in the mixtures using acetylene black compared with those for the mixtures using carbon black in this study, the surfactant and dispersant used in this study are more suitable for dissolving and dispersing carbon black than acetylene black.

The lowest resistance value of 2.64 Ω was observed at a carbon black to graphite weight ratio of 20%. For carbon black contents > 20%, there was an increasing trend of the resistance value as the content of the acetylene black mixture increased, similar to the results observed with acetylene black. These experimental results show that the ratio of carbon black used in inorganic EMP shielding paint ranged from 15% to 20% by weight compared with graphite.

### 3.3. Microstructural Analysis of EMP Shielding Paints

The materials (shielding raw materials, dispersants, binders, etc.) and content used in EMP shielding paint, as well as the manufacturing method, affect the dispersion state and interface properties of the carbon material, and also affect the EMP shielding effectiveness.

**Figure 7:**
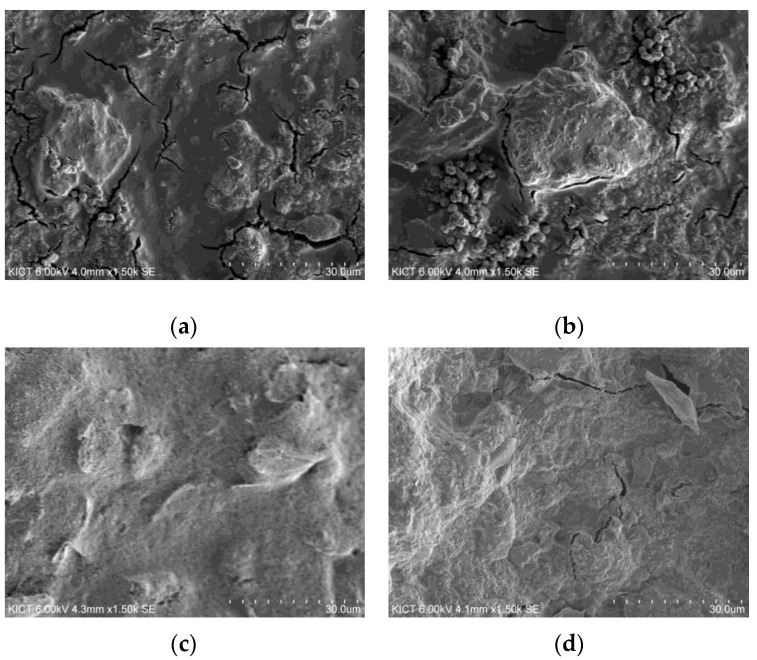
Microstructure analysis through SEM images: (**a**) Mix 2-2, (**b**) Mix 2-5, (**c**) Mix 2-8, (**d**) Mix 2-10.

The microstructure of the EMP shielding paint produced in the second mixing experiment was analyzed through scanning electron microscope (SEM) imaging. Through microstructure analysis, the dispersion state of the carbon material, interface characteristics, and microstructure inside the coating were identified. The microstructure analysis was conducted on the mixtures with the highest and lowest electrical resistance values among paints using acetylene black (mix 2-2, mix 2-5) and carbon black (mix 2-8, mix 2-10). SEM characterizations of each mixture of paints are presented in Figure 7. The magnification of the SEM images in Figure 7 is all 1500 times.

In Figure 7a,b, which are SEM characterization of paint using acetylene black, a cracked interface was observed. Cracks at the interface may prevent the flow of electrons, reducing the conductivity of EMP shielding paint. Therefore, the electrical conductivity is expected to be low [30], and the electrical resistance value was also measured to be high. In Figure 7b, it was confirmed that in the mixture of graphite and acetylene at a weight ratio of 1:0.3, acetylene black was not properly dispersed and was clumped in various places. This coagulation phenomenon can also be considered a cause of the reduced conductivity of the paint.

In Figure 7c, which is an SEM characterization of mix 2-8 with the lowest electrical resistance measured, cracks found in other mixtures were not observed. It was also confirmed that carbon black and graphite were well dispersed and there was no coagulation phenomenon. In Figure 7d, which is an SEM characterization of mix 2-10, cracks were observed. However, no coagulation of carbon black was found. As the amount of carbon black used increases, the amount of water and binder required for dispersion also increase. It is considered that the water and binder used in mix 2-10 were insufficient to disperse the carbon black and graphite. However, increasing the dosage of binder used can also cause a decrease in EMP shielding effectiveness. Therefore, finding the appropriate mixture proportions of each material through experimentation is important in improving EMP shielding effectiveness.

The electrical resistance value of a mixture using the same dosage of acetylene black was measured to be up to 15 times higher than that of a mixture using carbon black. One of the reasons is that, as mentioned in the previous section, the dispersant and surfactant used in this study were improper for acetylene black. Another reason is that the size of acetylene black particle is about 1/180 that of carbon black, and the specific surface area is relatively larger than that of carbon black. Therefore, it is considered that the amount of solvent required for dispersing acetylene black is relatively larger than that required for dispersing carbon black.

### 3.4. Evaluation of the Shielding Effectiveness of Inorganic EMP Shielding Paint

The performance of the inorganic EMP shielding paint at the optimal mix proportion (mix 2-8) was evaluated using the ASTM D 4935 test method [27]. The shielding effectiveness test was conducted by the authorized and certified Gumi Electronics & Information Technology Research Institute to ensure the fairness of the measurement results. The frequency range for evaluating the shielding effectiveness ranged from 30 MHz to 1.5 GHz. For detailed information on experimental equipment and methods, please refer to reference [27].

**Figure 8:**
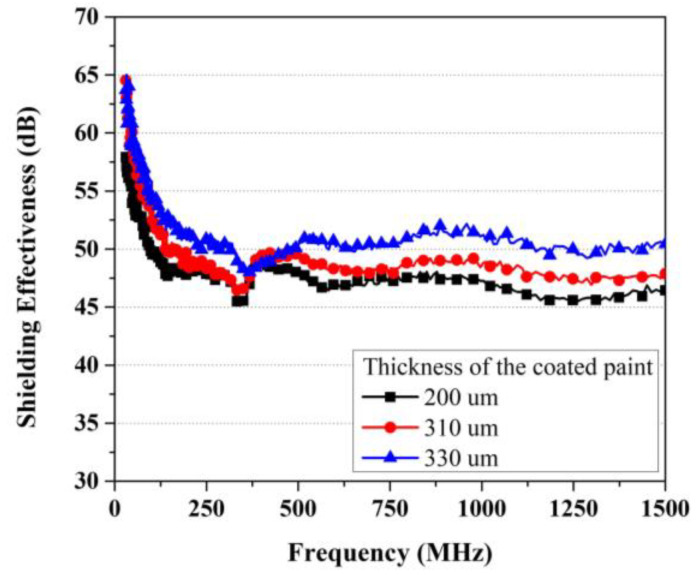
Results of EMP shielding effectiveness for mix 2-8.

The evaluation of shielding effectiveness was conducted for three types of paint with thicknesses equal to 200 μm, 310 um, and 330 μm; the results are shown in Figure 8. The evaluated results show that both the 200 μm and 310 μm shielding paints yielded performances > 40 dB. The highest shielding effectiveness was observed at a low frequency of 30 MHz, and the effectiveness gradually decreased as the frequency increased. The improvement in shielding effectiveness due to an increase in thickness ranged from approximately 2 dB to a maximum of 10 dB in almost all sections except the frequency range between 350–500 MHz.

### 3.5. Evaluation of the Adhesion Strength and the Resistance to Environmental Factors

The adhesion strength and the resistance to environmental factors of mix 2-8, which evaluated EMP shielding effectiveness, were evaluated.

**Figure 9:**
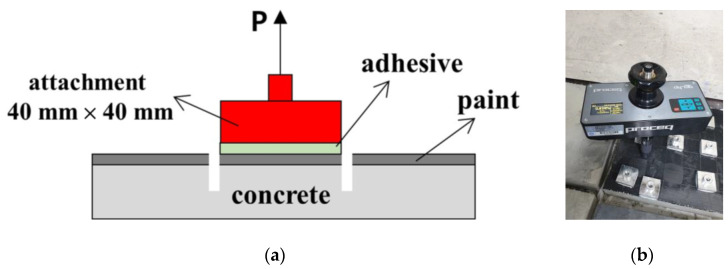
Evaluation of the bonding strength: (**a**) Schematic diagram of the bonding strength test and (**b**) Bonding strength test equipment and specimen.

The adhesion strength test was conducted according to the KS M 6010 [31] method, and the target strength was 0.5 MPa, which is the standard for KS M 6010 putty [31]. Figure 9 shows a schematic diagram of the adhesion strength test method and a photo of the experimental equipment. The test equipment can automatically control load and can perform tensile testing up to 10 MPa. The experiment was conducted with a load control speed of 0.28 MPa/s.

To perform the adhesion strength evaluation, a concrete panel with a size of 30 cm × 30 cm, a thickness of 5 cm, and a design strength of 24 MPa was manufactured. The concrete panel was cured for 14 days to develop compressive strength. The concrete panel surface to be painted was pretreated with a wire brush. After applying the EMP shielding paint and drying it for more than 24 h, a 40 mm × 40 mm steel attachment was installed. After setting the attachment, it was dried for more than 24 h to complete the production of the test specimen. The adhesion strength test was averaged by measuring six times per specimen according to the KS M 6010 test method [31].

Table 5 shows the results of the adhesion strength test. The average adhesion strength was 1.26 MPa; the maximum value was 1.72 MPa, and the minimum value was 0.92 MPa. It was found that the target adhesion strength of 0.5 MPa was satisfied.

**Figure 10:**
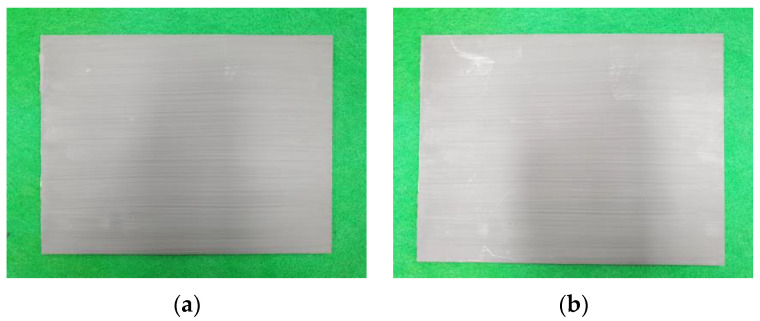
Specimens before and after the resistance test to the environmental factors: (**a**) Before test and (**b**) after test.

A moisture resistance evaluation was performed to evaluate the stability of the EMP shielding paint against external environmental factors (temperature, humidity). The moisture resistance test was conducted according to the KS D 8502 [32] method. The conditions for the moisture resistance test were a temperature of 50 ± 3 °C and a relative humidity of 95% or more. After being stored for more than 120 h under test conditions, the condition of the specimen was visually observed. Figure 10 shows photos of the specimen before and after the moisture resistance test. As a result of observing the surface of the specimen after being left for more than 120 h at a temperature of 50 ± 3 °C and a relative humidity of 95% or higher, no changes such as cracks and peeling were observed.

## 4. Shielding Effectiveness Verification Experiment for the Inorganic EMP Shielding Paint

### 4.1. Fabrication of Specimens

The shielding effectiveness of mix 2-8 paints, which were prepared by mixing graphite, carbon black, and inorganic binder (selected based on a mixing experiment), was evaluated on shielding concrete wall components. The specimen was a concrete wall measuring 2.2 m × 2.2 m × 0.1 m, which was fabricated based on a previous study [24]. Before applying the shielding paint, the concrete surface was sanded to remove dust. Subsequently, void and flattening work was performed using a putty, followed by two rounds of painting using a spray method. The thickness of the coated film was measured to be 200 μm on average. The measurement was conducted by performing spray coating on a metallic substrate under the same conditions as when producing test specimens during the mixing experiment.

**Figure 11:**
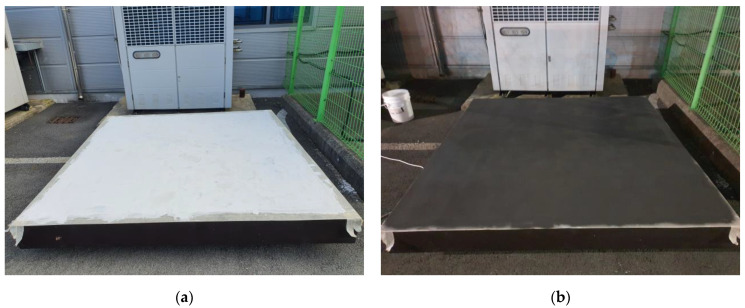
Specimen for EMP shielding effectiveness evaluation: (**a**) Specimen with putty and (**b**) specimen with EMP shielding paint.

Figure 11 is a specimen that was surface-treated with putty and then entirely coated with shielding paint. During both the putty and repetitive painting processes, a minimum drying time of at least 2 h was allowed before proceeding with the work. After the completion of the painting process, a sufficient drying time of 1 day was given to minimize the impact of moisture within the coating on the evaluation of shielding effectiveness.

### 4.2. Experimental Method

An evaluation of the shielding effectiveness was conducted on concrete specimens with EMP shielding inorganic paint applied to their walls. The experimental method adhered to the United States military standard MIL-STD-188-125 [33], and the shielding effectiveness was measured in the frequency range of 10 kHz–2.0 GHz.

**Figure 12:**
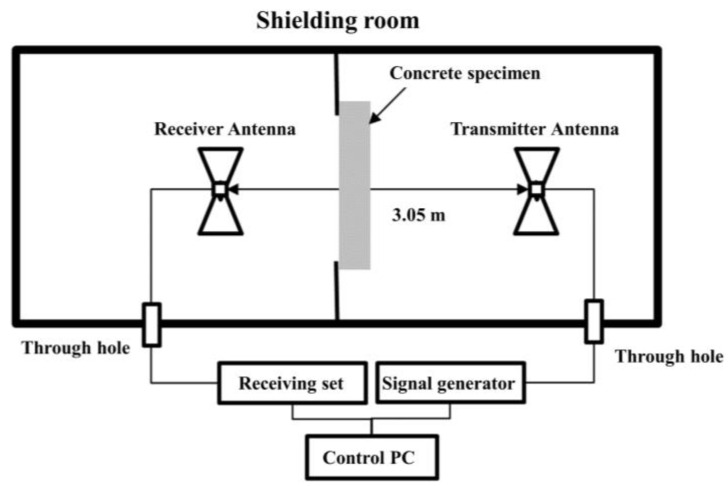
EMP shielding effectiveness test concept.

**Figure 13:**
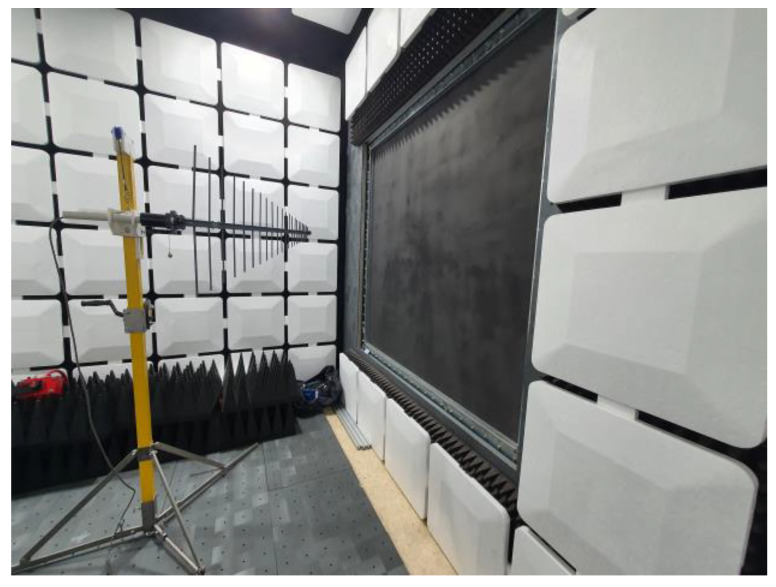
Appearance of an evaluated specimen during the EMP shielding effectiveness test.

Figure 12 depicts a schematic of the EMP shielding effectiveness test for large-scale concrete wall specimens. A concrete specimen was placed in the center, the transmitting and receiving antennas were placed approximately 3 m apart, and the shielding effectiveness was measured by using a signal generator (model SMBW100, Rohde & Schwarz). Loop, biconical, and log-periodic antennas were used for measurements depending on the frequency range. The measurement frequency ranges were 10 kHz–20 MHz, 20–300 MHz, and 300 MHz–2 GHz, respectively. Figure 13 illustrates the actual measurement of the shielding effectiveness using test specimens and antennas.

### 4.3. EMP Shielding Effectiveness Evaluation Results

**Figure 14:**
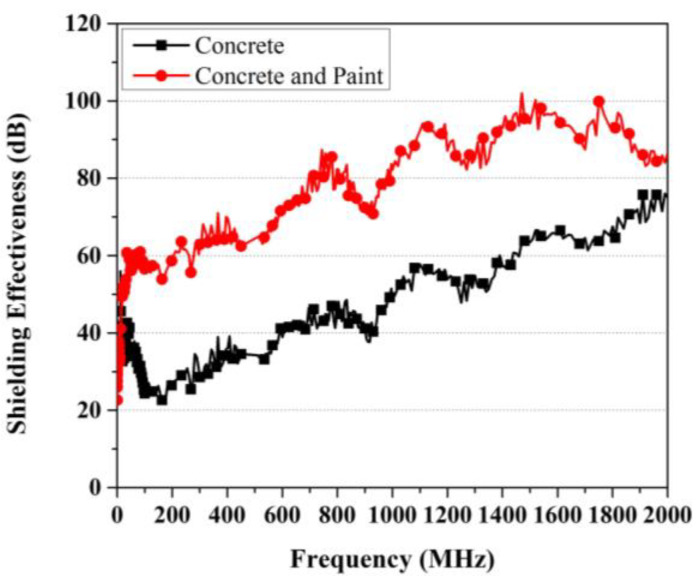
Evaluation results of EMP shielding effectiveness for large concrete wall specimens.

The evaluation results of the shielding effectiveness of concrete specimens before and after the application of paint are presented in Figure 14. The results of the shielding effectiveness evaluation revealed that the concrete specimens without the application of EMP shielding inorganic paint exhibited shielding effectiveness ranging from approximately 20 dB to a maximum of 75 dB across different frequency ranges. However, when the paint was applied, the shielding effectiveness improved by approximately 25 dB up to 43 dB. The results indicate an increase in shielding effectiveness greater than the reported 10–15 dB improvement for a 100 mm increase in concrete thickness, as noted in the previous study [11]. These experimental results confirmed that applying the EMP shielding inorganic paint with a small thickness can enhance the shielding effectiveness by 40 dB or more.

Examining the overlapping effects of EMP shielding materials revealed that the maximum shielding effectiveness of each material in this experiment did not overlap across all frequency ranges. It is believed that there was a loss of shielding effectiveness owing to the presence of an interface, indicating that the concrete and paint were not completely bonded together. This result pertains specifically to the materials used in this experiment and does not apply to the overlapping effects of other shielding materials.

This study verified the feasibility of applying EMP shielding inorganic paints to buildings. Additional research will be conducted to validate its application through mock-up experiments and actual applications to structures in the future.

## 5. Conclusions

Mixing experiments were conducted on 27 types of paint with different shielding materials and binders to develop an EMP shielding inorganic paint using carbon materials. The shielding effectiveness of EMP shielding paint for building interiors was evaluated, and adhesion strength and moisture resistance were also evaluated. The conclusions obtained from this study are as follows.

1. To select the most suitable binder for EMP shielding inorganic paint, mixture experiments were conducted using four types of binder. It was confirmed that an inorganic binder produced by modifying sodium-based water glass and potassium-based water glass, rather than using them alone, was effective in improving the shielding performance of the paint.
2. As a result of measuring the resistance value of the shielding paint according to the type of additive, the resistance value of carbon black was significantly lower than that of acetylene black. The electrical resistance value of a mixture using the same dosage of acetylene black was measured to be up to 15 times higher than that of a mixture using carbon black.
3. According to the measured electrical resistance values based on the weight ratio of carbon black in graphite, the resistance value increased as the carbon black content increased for contents ≥ 20%. The ratio of carbon black used in inorganic EMP shielding paint was estimated to be in the range of 15–20% by weight of graphite.
4. To verify the shielding effectiveness improvement effect of EMP shielding paint for building interiors, it was applied to shielding concrete. As a result, the application of EMP shielding inorganic paint improved the shielding effectiveness by approximately 25 dB and up to 43 dB.
5. The adhesion strength and moisture resistance evaluation of the EMP shielding paint were evaluated. The average adhesive strength of the EMP shielding paint was 1.26 MPa. As a result of a moisture resistance test left at a temperature of 50 ± 3 °C and a relative humidity of 95% or higher for more than 120 h, no cracks or peeling were observed on the painted surface.

The EMP shielding paint for building interiors developed in this paper can be used as basic data to improve the EMP shielding performance of structures in the future.

## Figures and Tables

**Table 1:** Physical properties of carbon materials.

Content	Graphite	Acetylene Black	Carbon Black
carbon content (%)	96–99.9	>95	>95
particle size (μm)	2.0–7.0	0.035	6.5–12.5
bulk density (g/L)	2230	250	112

**Table 2:** Specifications of binder materials.

Product	PH	Specific Gravity (20 ± 1 °C)	Nonvolatile Content (wt %, 20 ± 1 °C, 1 h)	Viscosity (cps, 20 ± 1 °C)
sodium silicate	12–13	1.38	-	100
potassium silicate	11–12	1.33	-	50
inorganic binder	11–12	1.3–1.4	37 ± 1	200–400
nanohybrid resins	9–10	1.1–1.2	43 ± 1	50

**Table 3:** Mixture proportions and electrical resistance measurement results of experimental series 1 (main shielding material: graphite).

Mixture Weight (g)							
Mixture (mix)	Graphite	Water	Sodium Silicate	Potassium Silicate	Inorganic Binder	Nanohybrid Resin	Electric Resistance (Ω)
mix 1-1	200	200	-	-	-	-	5.54
mix 1-2	200	-	200	-	-	-	7.91
mix 1-3	200	-	-	200	-	-	3.43
mix 1-4	200	-	-	-	200	-	2.22
mix 1-5	200	-	-	-	-	200	64.5
mix 1-6	200	140	100	-	-	-	12.4
mix 1-7	200	140	200	-	-	-	16.6
mix 1-8	200	140	300	-	-	-	17.8
mix 1-9	200	140	400	-	-	-	25.8
mix 1-10	200	140	-	100	-	-	11.4
mix 1-11	200	140	-	200	-	-	8.46
mix 1-12	200	140	-	300	-	-	10.4
mix 1-13	200	140	-	400	-	-	12.5
mix 1-14	200	140	-	-	100	-	8.48
mix 1-15	200	140	-	-	200	-	2.47
mix 1-16	200	140	-	-	300	-	5.49
mix 1-17	200	140	-	-	400	-	23.4

**Table 4:** Mixture proportions and measured electrical resistance results.

Mixture Weight (g)						
Mixture	Graphite	Acetylene Black	Carbon Black	Inorganic Binder	Water	Electric Resistance (Ω)
mix 2-1	200	20	-	200	400	29.6
mix 2-2	200	30	-	200	440	21.3
mix 2-3	200	40	-	200	460	29.1
mix 2-4	200	50	-	200	480	55.3
mix 2-5	200	60	-	200	500	66.4
mix 2-6	200	-	20	200	400	5.25
mix 2-7	200	-	30	200	440	4.31
mix 2-8	200	-	40	200	460	2.64
mix 2-9	200	-	50	200	480	7.21
mix 2-10	200	-	60	200	500	7.94

**Table 5:** Results of the adhesion strength test.

Mix	Adhesion Strength (MPa)						
	#1	#2	#3	#4	#5	#6	Average
Mix 2-8	0.92	1.72	1.65	1.41	1.07	1.32	1.26

## Data Availability

Data are contained within the article.
